# High-energy visible light transparency and ultraviolet ray transmission of metallized rescue sheets

**DOI:** 10.1038/s41598-019-47418-8

**Published:** 2019-08-01

**Authors:** Markus Isser, Hannah Kranebitter, Erich Kühn, Wolfgang Lederer

**Affiliations:** 1 Medical Division, Mountain Rescue Tyrol, Telfs, 6410 Austria; 2 Medical University of Innsbruck, Department of Anesthesiology and Critical Care Medicine, Innsbruck, 6020 Austria; 3 Private Polytechnic Institute of the State of Tyrol - College of Optometry, Hall, 6060 Austria

**Keywords:** Environmental biotechnology, Medical research, Preclinical research, Mechanical properties, Optics and photonics

## Abstract

Metallized rescue sheets are essential components in first aid boxes and professional emergency equipment for provision of thermal insulation. We investigated the transparency for visual light and the transmission of ultraviolet radiation and high-energy visible light in the violet/blue band of rescue sheets under laboratory conditions to evaluate the potential of blocking solar radiation during outdoor activities. An experimental study was performed using two commercially available brands of rescue sheets. Transmission of visible light and ultraviolet light was assessed by optometry. Single-layer transparency for visible light was between 1% and 8%. Transmission for high-energy visible light in the violet/blue band and ultraviolet A rays was between 1% and 13% for the single layer and between 0% and 3% for the double layer of the rescue sheets. Transmission for ultraviolet B rays afforded by each tested rescue sheet brand was between 0% and 1% for the single layer. Double-layer rescue sheets blocked 100% of ultraviolet B radiation. In conclusion, single layer rescue sheets were sufficiently permeable for visible light and diminished transmission for ultraviolet radiation and high-energy visible light in the violet/blue band to potentially protect from solar radiation if used for facial protection and as makeshift sun googles.

## Introduction

Damage to eyes and skin from solar radiation are mostly associated with high-energy rays in the ultraviolet (UV) spectrum and high-energy visible (HEV) light in the violet/blue band^1–4^. While ultraviolet C (UVC) rays are blocked by the ozone layer of the atmosphere, ultraviolet B (UVB) and mostly ultraviolet A (UVA) rays penetrate the atmosphere^5^. Numerous factors influence sun exposure hazards, including solar elevation, time of day and season, atmospheric conditions, climate, geographic location and altitude, ground reflection and diffuse radiation, age, gender and behavior, skin phototypes, chemical photosensitivity, among others^5–8^. Increased likelihood of contracting ocular damage from lasting solar radiation may occur even while wearing sunglasses if radiant energy from the side and from around the frame can reach the eye^4,7,9,10^.

Acute eye effects include snow blindness and retinal burn. Painful injuries of cornea and conjunctiva from intense solar radiation reflected by snow or glaciers are well known medical emergencies^4,8,11,12^. Acute photokeratitis from short-term exposure to high doses of UVB rays was reported in up to 3% of high-altitude mountaineers^8,11^. The UV exposure threshold for photokeratitis was reported to be less than 10 minutes if reflected from a horizontal ground surface around midday in the summer sun^9^. Solar retinopathy may result from thermal damage of the retina related to visible light, in particular blue light around 500 nm of wave length and infrared components of solar UV radiation^4^. Eye effects related to chronic sunlight exposure include photochemical cataract and pterygium, due to UV component, uveal melanoma and eyelid malignancies, and macular degeneration related to visible light and near UVA^2–4^. Acute skin effects of solar radiation include sunburn and photodermatoses while chronic exposure may initiate the development of actinic keratoses, photoaging, and skin cancers^4,13^.

Contrasting qualities are typical for multi-functional rescue sheets. The gold color and light reflection from the surface increase visibility for search and rescue services but wrapping the body in a rescue sheet diminishes visibility for infrared detectors^14^. On the one hand, the watertight and windproof foils provide thermal insulation and prevent hypothermia by reducing thermo-convection and diminishing heat loss from evaporation and thermal radiation^15–17^. On the other hand, the sheets promote cooling by acting as a radiant barrier, by providing shade and even by increasing heat conduction when the sheet is in direct contact with the skin. Although there are reports that rescue blankets can reflect infrared radiation, so far, no information is provided on transmission of UV radiation and HEV-light^14^.

In an experimental study we investigated the degree of visible light transparency and ultraviolet ray transmission of conventional rescue sheets commonly used by emergency medical services. We wanted to find out whether rescue sheets can block solar radiation and function as makeshift snow goggles in the absence of sunglasses.

## Results

Two commercially available rescue sheets (Mountain Rescue Tyrol (MRT) Rescue Blanket; Austrian Red Cross (ARC) Rescue Sheet) were tested. The two sheets differed slightly regarding transparency of visible light and transmission of UV radiation and HEV light in the violet/blue band.

### Measurement of transparency for visible light

**Figure 1:**
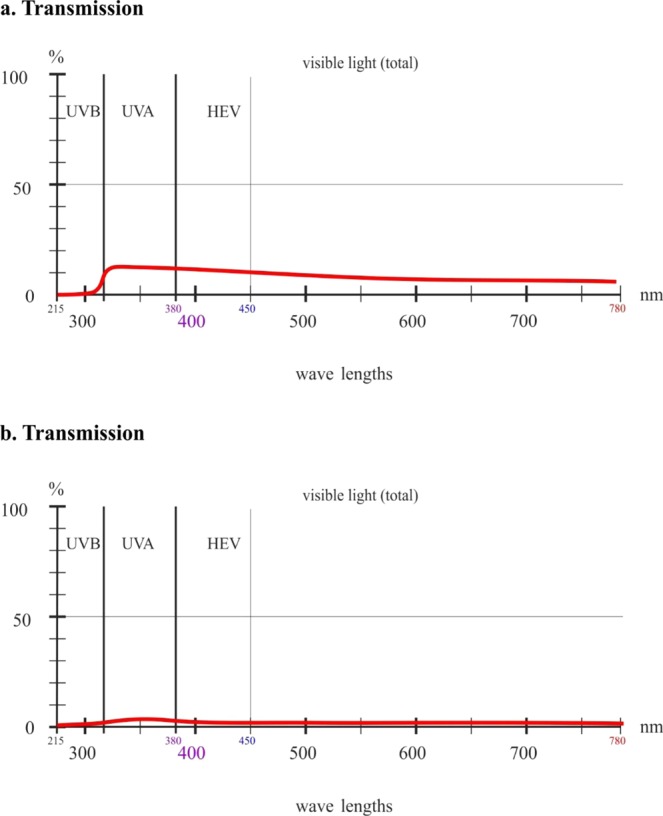
Transmission of ultraviolet light B (UVB) and ultraviolet light A (UVA) and transparency for high-energy visible (HEV) light in the violet/blue band and visible light through single-layer (**a**) and double-layer (**b**) MRT Rescue Blankets.

**Figure 2:**
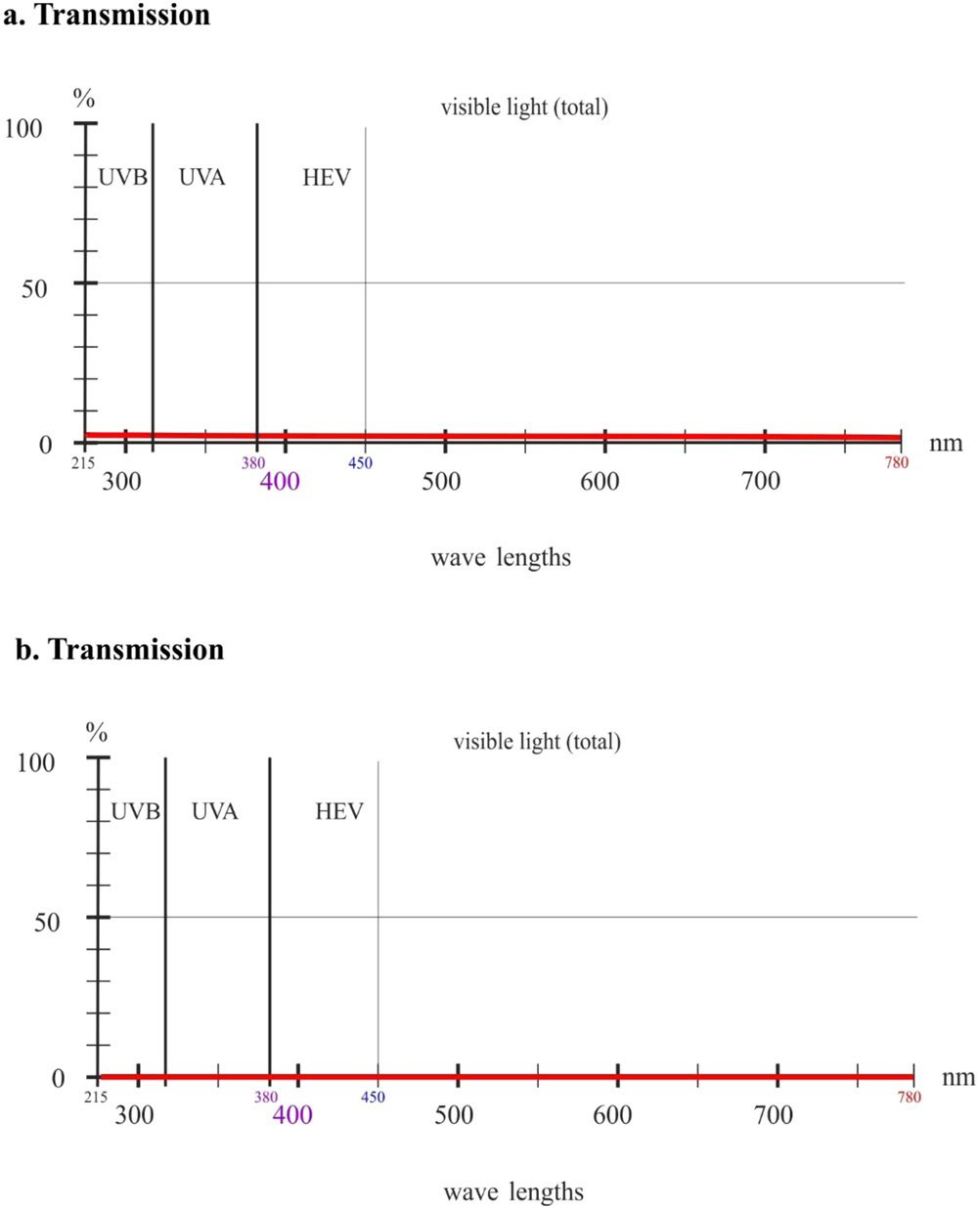
Transmission of ultraviolet light B (UVB) and ultraviolet light A (UVA) and transparency for high-energy visible (HEV) light in the violet/blue band and visible light through single-layer (**a**) and double-layer (**b**) ARC Rescue Sheets.

Visible light transmission for the single layer of the MRT Rescue Blanket was 8% measured at 550 nm, while for the double layer of the sheet it was 1% (Fig. 1). Transmission for the single layer of the ARC Rescue Sheet was 1% for visible light and 0% for the double layer of the blanket (Fig. 2). Transparency did not depend on the color of the exposed surface, namely silver or gold, in either brand.

### Measurement of transmission of UV light and HEV light

The two tested rescue sheet brands blocked between 99% and 100% of ultraviolet B radiation. Transmission for the single layer of the MRT Rescue Blanket was 1% for UVB, 13% for UVA up to 380 nm, 12% for long-wave UVA in the UV/visible radiation boundary region and 10% for HEV light in the violet/blue band. Transmission for the double layer of the sheet was 1% for UVB, 3% for UVA up to 380 nm, 2% for long-wave UVA in the UV/visible radiation boundary region and 1% for HEV light in the violet/blue band (Fig. 1). Transmission for the single layer of the ARC Rescue Sheet was 1% for UVB and 1% for short- and long-wave UVA and HEV light in the violet/blue band. Transmission for the double layer of the sheet was 0% for UV radiation and HEV light (Fig. 2). Even for the single layer between 99% and 100% of UVB light was blocked. No differences were detected in the two brands when the exposed surface color was silver or gold.

### Feasibility *in vivo*

**Figure 3:**
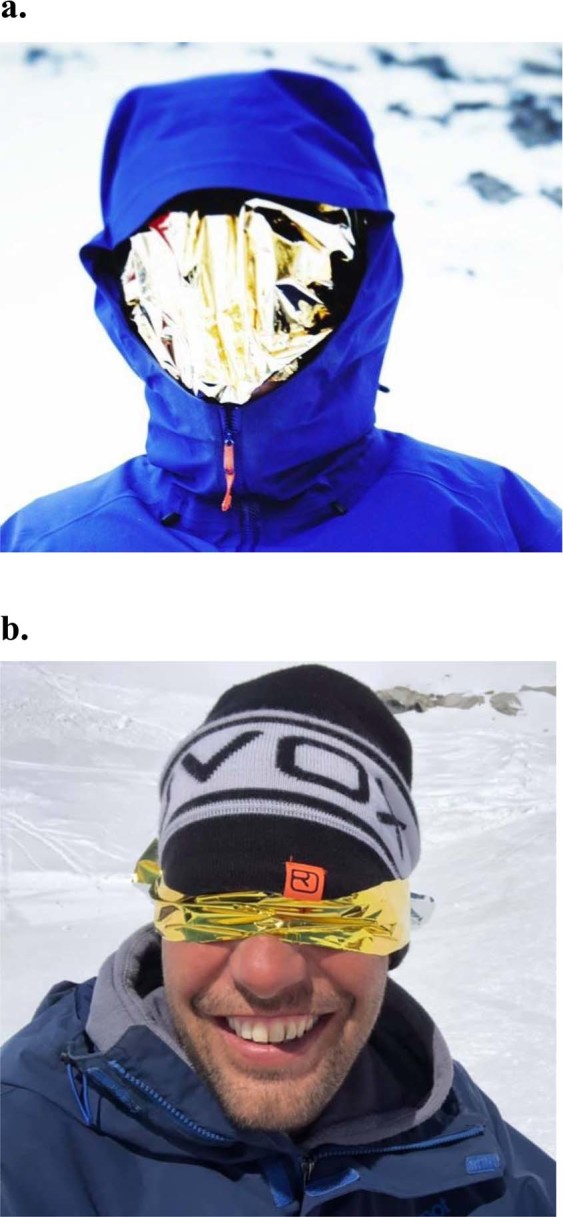
Transparency of metallized rescue sheets. Facial protection (**a**) and makeshift sun goggles (**b**) made of a commercially availabe single layer rescue sheet. The techniques of application were demonstrated by two Austrian mountain guides as the models.

Both, facial protection and makeshift sun goggles, were easily established with a single layer rescue sheet and tested by four volunteers (Fig. 3). One volunteer of Austrian Mountain Rescue even used makeshift goggles of a single layer rescue sheet during his assistance in a real avalanche emergency for more than three hours. Despite exposure to high solar irradiation around midday in high altitude none of the rescue personnel suffered ocular impairment. However, although sun cream was applied most of them contracted sunburn in the neck.

## Discussion

We here report on eye and skin protective properties of multi-functional metallized rescue sheets generally used in first aid and emergency medicine. In our experimental investigation single-layer rescue sheets of both tested brands were sufficiently permeable for visible light. In addition, they adequately blocked transmission for UV rays and HEV light in the violet/blue band to potentially protect from solar radiation. Transmission for ultraviolet B rays afforded by each tested rescue sheet brand was between 0% and 1% for the single layer. As there is still sufficient transparency for visible light, a single layer rescue sheet put over the mountaineer’s head allows him to descend under his own power while being protected from ultraviolet radiation (Fig. 3). The International Commission for Mountain Emergency Medicine (ICAR-MEDCOM) recommends that any sight-threatening eye problem or unexplained visual loss at high altitude necessitates descent^7^. However, acute pain, blepharospasm, increased flow of tears and temporary loss of vision can make further climbing impossible. Furthermore, bad weather conditions can even frustrate rapid rescue and delayed treatment is common in remote emergency locations.

No doubt that protection from UV radiation at high altitudes is of utmost importance but when proper snow goggles are lacking rescue sheets have at least the potential to temporarily protect from snow blindness. Then again, protection from HEV light during alpine sport activities is given low priority though extended exposure to HEV light may be hazardous to the eye and be associated with blurred vision and affected visual contrast^3^. When investigating double-layer rescue sheets only the ARC brand provided limited transparency for visible light. The double-layer MRT brand had no transparency for UV radiation, HEV light and visible light thus, providing absolute blackout for a patient with acute photokeratitis awaiting extrication. Sunburn following excessive exposure to UV radiation results of a phototoxic effect in the skin^6^ and may be extremely harmful at high altitude. In particular, absorption of DNA is detected for wavelengths shorter than 325 nm^6^. The International Commission on Non-Ionizing Radiation Protection recommended that protection by sunscreens should be a protective measure only when other measures are not practical^6^. However, in the absence of adequate preventive measures rescue sheets might provide temporary protection from solar radiation during outdoor activities.

We conducted an experimental study on protective properties against UV radiation and HEV light of two commercially available recue sheets but there are several limitations to be considered. We are aware that thickness and transparency of foils can differ between production series and among the variety of available rescue sheet products and may have direct impact on transparency. We did not use a modelling tool to estimate exposure of eye and different skin zones under rescue sheets to predicted UV doses^7^ nor did we test protective effects on tissue *in vitro*. So far, we could gather information on the eye protective effect of metallized rescue sheets on ultraviolet B rays only in a single actual emergency. Furthermore, we cannot tell whether metallized sheets potentially prevent solar retinopathy as we did not evaluate the infrared components of solar UV radiation. In our study we investigated only the applicability of using a metallized rescue sheet as makeshift sun goggles or facial protection taking into account that rescue sheets for facial protection must not be kept tight to the skin in order to avoid carbon dioxide rebreathing. We investigated the percentage of relative transmission but not the accurate protection from UV radiation by makeshift goggles made of a single layer rescue sheet under high solar irradiation conditions. On the one hand, high levels of solar radiation may lead to pupillary constriction and squinting that reduce ocular exposure^6^. On the other hand, rescue sheets block UV radiation form the side but protective effects are diminished if radiant energy from ground reflection can reach the eye. This is comparable with wearing conventional sunglasses^4,9,10^. However, there is need to prospectively test the protective functions of makeshift sun goggles against snow blindness during outdoor activities under high solar irradiation.

In conclusion, our findings presumably increase the range of multi-functional uses of rescue sheets by one more application. If the eye protective effect of metallized rescue sheets can supplement the multi-functional scope of rescue sheets in the recommendations of ICAR MEDCOM, the degree of UV protection has to be investigated and recorded on the package of each commercially available rescue sheet.

## Materials and Methods

An experimental study on commercially available rescue sheets regarding transparency for visible light and UV radiation was performed. Transmission of direct UV radiation and HEV light was tested under controlled conditions in the laboratory. Applicability *in vivo* using a metallized rescue sheet as makeshift snow goggles or facial protection was tested in four volunteers (Fig. 3). Rescue sheets are medical devices category 1 following the directive 93/42/EEC^18^. According to the proposed new ocular traumatology terms the study focuses on prevention of an injury to the eye wall involving sclera and cornea^19^.

### Ethical approval and volunteer consent

Informed consent for both study participation and publication of identifying information/images in an ‘online open-access publication’ was obtained from two volunteers of Austrian Mountain Rescue and two Austrian mountain guides who used rescue sheets as makeshift goggles. The experimental study has been complied with all the relevant national regulations, institutional policies and is in accordance with the tenets of the Helsinki Declaration^20^. Ethics approval for an experimental study not involving patients was not required according to the committee’s officers of the institutional ethics committee. The authors confirm that our institutional ethics committee formally exempted our study from requiring approval of our study protocols.

### Experiment design

Transparency for visible light and HEV light in the violet/blue band, as well as the transmission of short- and long-wave UVA and UVB were measured. We investigated two different rescue sheets commonly used by either Mountain Rescue and Helicopter EMS (MRT Rescue Blanket, REF 43000) or ground Emergency Medical Services (EMS) of Red Cross (ARC Rescue Sheet, 2015265). Both rescue sheets were 160 × 210 cm in size and 0.012 mm in thickness, 1% aluminium coated, with surface colour silver or gold on either side.

### Measurement of permeability for visible light and protection from UV light and HEV light

Transmission of direct radiation at wavelengths between 215 nm and 780 nm was measured optometrically using a lens analyzer (Humphrey Systems LA 360; Carl Zeiss Meditec Inc., Dublin, CA, USA). Light visible to the human eye was defined at wavelengths between 380 nm and 740 nm^21^. Results were displayed as transmission curve of wavelengths of UVB from 280 to 315 nm, of UVA in the non-visible light from 315 to 380, long-wave UVA in the UV/visible radiation boundary region from 380 to 400 nm^13^ and of HEV light in the violet/blue band from 400 to 450 nm. Degree of transmission of UV radiation was indicated in percent: see supplementary information on permeability for visible light and protection from UV light and HEV light.

### Statistical analysis

Descriptive statistics was applied using univariate analysis of transmission to provide summaries and graphs. Median was expressed as measure of central tendency and standard deviation as measures of variability. Minimum and maximum values were expressed as range.

## Supplementary information

LaTeX Supplementary File

## References

[1] Young, R. W. The family of sunlight-related eye diseases. *Optom*. *Vis*. *Sci*. **71**, 125–144, PMID: 8152745 (1994). 10.1097/00006324-199402000-00013 8152745

[2] Lucas, R., McMichael, T., Smith, W. & Armstrong, B. Solar and ultraviolet radiation – Global burden of disease from solar ultraviolet radiation. World Health Organization, Geneva 2006, WHO, https://www.who.int/uv/publications/solaradgbd/en/ (2019).

[3] Yam JC, Kwok AK (2014). Ultraviolet light and ocular diseases. Int. Ophthalmol..

[4] Modenese Alberto, Korpinen Leena, Gobba Fabriziomaria (2018). Solar Radiation Exposure and Outdoor Work: An Underestimated Occupational Risk. International Journal of Environmental Research and Public Health.

[5] Schmalwieser AW (2010). Facial Solar UV Exposure of Austrian Farmers During Occupation. Photochem. Photobiol..

[6] International Commission on Non-Ionizing Radiation Protection (2010). ICNIRP statement–Protection of workers against ultraviolet radiation. Health Phys..

[7] Backes, C. *et al*. Sun exposure to the eyes: predicted UV protection effectiveness of various sunglasses. *J*. *Expo*. *Sci*. *Environ*. *Epidemiol*., 10.1038/s41370-018-0087-0 (2018). 10.1038/s41370-018-0087-0 PMC6803516 30382242

[8] Izadi M, Jonaidi-Jafari N, Pourazizi M, Alemzadeh-Ansari MH, Hoseinpourfard MJ (2018). Photokeratitis induced by ultraviolet radiation in travelers: A major health problem. J. Postgrad. Med..

[9] Sliney DH (2002). How light reaches the eye and its components. Int. J. Toxicol..

[10] Notara M (2018). light-blocking contact lenses protect against short-term UVB-induced limbal stem cell niche damage and inflammation. Sci. Rep..

[11] Basnyat, B. & Litch, J. A. Medical problems of porters and trekkers in the Nepal Himalaya. *Wilderness*. *Environ*. *Med*. **8**, 78–81, PMID: 11990146 (1997). 10.1580/1080-6032(1997)008[0078:mpopat]2.3.co;2 11990146

[12] Ellerton JA, Zuljan I, Agazzi G, Boyd JJ (2009). Eye problems in mountain and remote areas: prevention and onsite treatment-official recommendations of the International Commission for Mountain Emergency Medicine ICAR MEDCOM. Wilderness. Environ. Med..

[13] Lawrence KP (2018). The UV/Visible Radiation Boundary Region (385-405 nm) Damages Skin Cells and Induces “dark” Cyclobutane Pyrimidine Dimers in Human Skin *in vivo*. Sci. Rep..

[14] Jorgustin K. How To Block IR Infrared Thermal Imaging. Modern Survival Blog, https://modernsurvivalblog.com/security/how-to-block-ir-infrared-thermal-imaging/ (2019).

[15] Chadwick, S. & Gibson, A. Hypothermia and the use of space blankets: a literature review. *Accid*. *Emerg*. *Nurs*. **5**, 122–125, PMID: 9325662 (1997). 10.1016/s0965-2302(97)90001-1 9325662

[16] Henriksson, O., Lundgren, J. P., Kuklane, K., Holmér, I. & Bjornstig, U. Protection against cold in prehospital care-thermal insulation properties of blankets and rescue bags in different wind conditions. *Prehosp*. *Disaster*. *Med*. **24**, 408–415, PMID: 20066643 (2009). 10.1017/s1049023x00007238 20066643

[17] Haverkamp FJC, Giesbrecht GG, Tan ECTH (2018). The prehospital management of hypothermia - An up-to-date overview. Injury..

[18] Council Directive 93/42/EEC of 14 June 1993 concerning medical devices, https://cemarking.net/medical-devices-directive/ (2019).

[19] Kuhn, F. *et al*. A standardized classification of ocular trauma. *Graefes Arch*. *Clin*. *Exp*. *Ophthalmol*. **234**, 399–403, PMID: 8738707 (1996). 10.1007/BF00190717 8738707

[20] World Medical Association (2013). Declaration of Helsinki: ethical principles for medical research involving human subjects. JAMA..

[21] Space Environment Technologies. ISO 21348 Definitions of Solar Irradiance Spectral Categories, https://www.spacewx.com/pdf/SET_21348_2004.pdf (2019).

