# Supplementary material for: High-energy visible light transparency and ultraviolet ray transmission of metallized rescue sheets
Source: Sci Rep. 2019 Aug 1;9:11208. doi: 10.1038/s41598-019-47418-8 (PMC6671957; doi:10.1038/s41598-019-47418-8)
Supplement: Supplementary file 1 — LaTeX Supplementary File [file 41598_2019_47418_MOESM1_ESM.pdf]

High-energy visible light transparency and ultraviolet ray transmission of metallized rescue sheets

Markus Isser[1], Hannah Kranebitter[1,2], Erich Kühn[3,+], Wolfgang Lederer[2,+,*]

[1] Medical Division, Mountain Rescue Tyrol, Telfs, 6410, Austria.

[2] Medical University of Innsbruck, Department of Anesthesiology and Critical Care Medicine, Innsbruck, 6020, Austria

[3] Private Polytechnic Institute of the State of Tyrol - College of Optometry, Hall, 6060, Austria.

We investigated two different rescue sheets commonly used by either ground Emergency Medical Services (EMS) of Red Cross (ARC Rescue Sheet, 2015265) or Mountain Rescue and Helicopter EMS (MRT Rescue Blanket, REF 43000). Both rescue sheets were 160 x 210 cm in size and 0.012 mm in thickness, 1% aluminium coated, with surface colour silver or gold on either side.

Transparency for visible light and HEV light in the violet/blue band, as well as the transmission of short- and long-wave UVA and UVB were measured. Transmission of direct radiation at wavelengths between 215 nm and 780 nm was measured optometrically using a lens analyzer (Humphrey Systems LA 360; Carl Zeiss Meditec Inc., Dublin, CA, USA). Light visible to the human eye was defined at wavelengths between 380 nm and 740 nm**.** Results were displayed as transmission curve of wavelengths of UVB from 280 to 315 nm, of UVA in the non-visible light from 315 to 380, long-wave UVA in the UV/visible radiation boundary region from 380 to 400 nm and of HEV light in the violet/blue band from 400 to 450 nm.

UVC defined at wavelengths between 100 nm and 215 nm rays are not indicated as they are blocked by the ozone layer of the atmosphere.

The red line in the graph below indicated the transmission curve. Degree of transmission of UV radiation was indicated in percent.

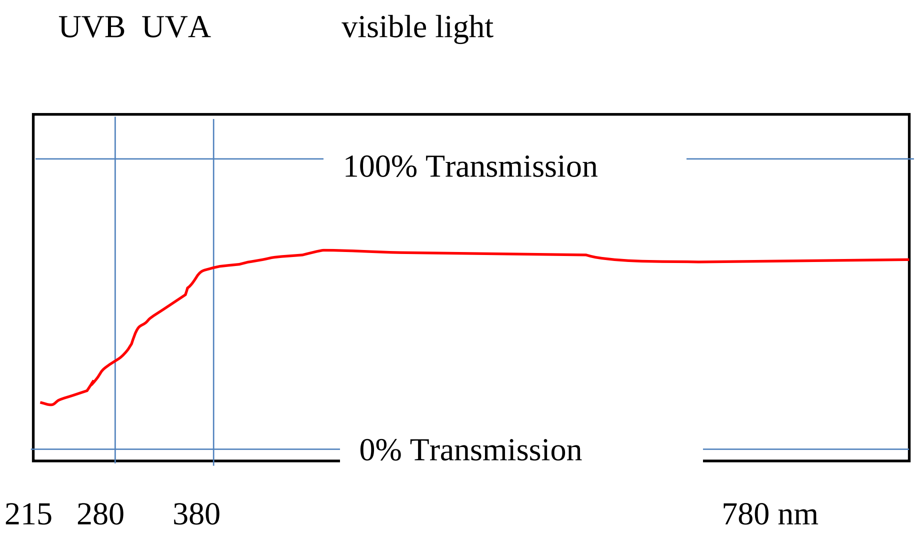

ARC Rescue Sheet – single layer

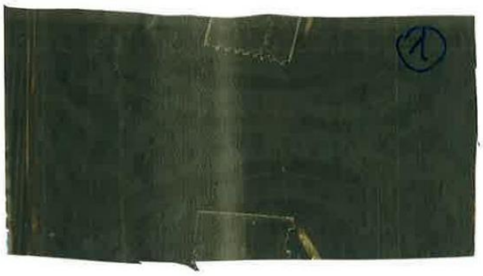

Transmission:

Control / no rescue sheet:

100 % transmission for UV- and visible light

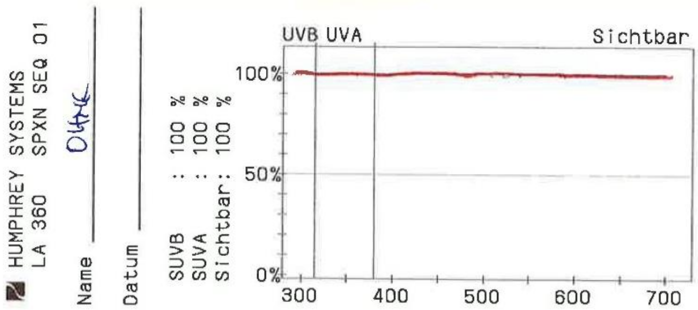

ARC Rescue Sheet – single layer silver/gold:

Minimal transmission (1%) for UVA, UVB- and visible light

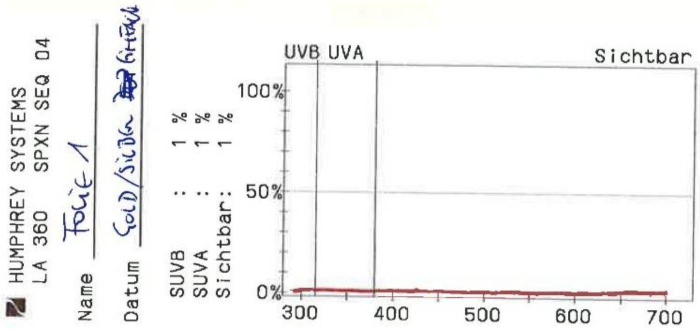

ARC Rescue Sheet – double layer

No transmission for UV- and visible light!

This implies the potential of 100% protection from UV and visible light
and absolute blackout

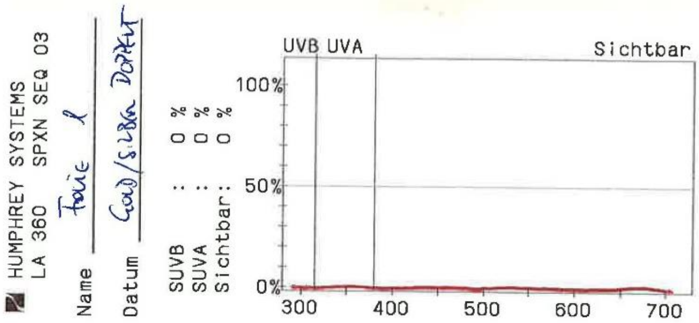

MRT Rescue Blanket – single layer

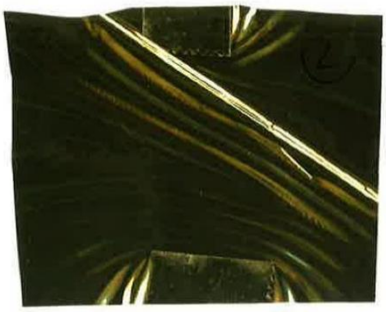

Transmission:

Control / no rescue sheet:

100 % transmission for UV- and visible light

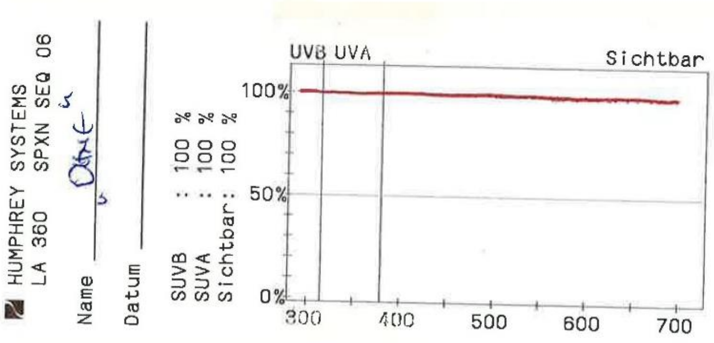

MRT Rescue Blanket – single layer silver:

Minimal transmission (1%) for UVB

Comparatively high transmission for UVA (13%)

Diminished transmission for visible light (8%) → but sufficient blackout

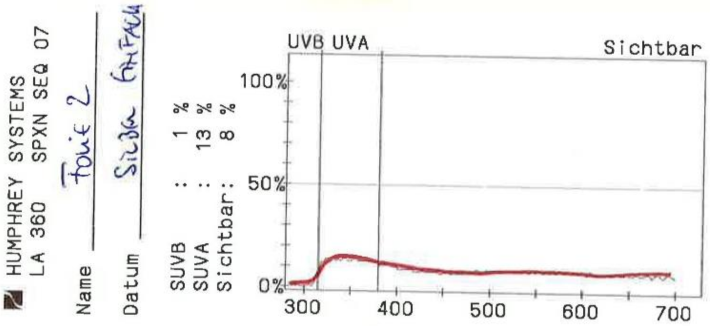

MRT Rescue Blanket – single layer gold:

Results are comparable regardless whether exposed surface was silver or gold!

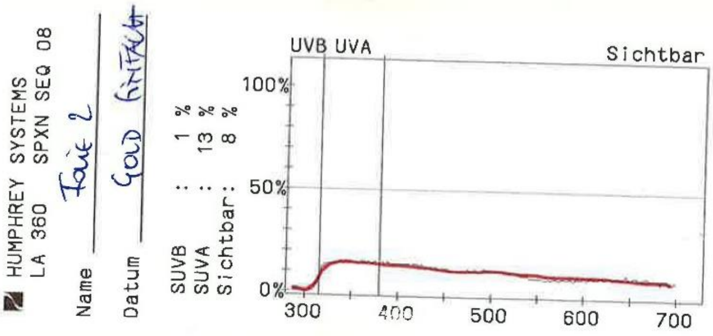

MRT Rescue Blanket – double layer

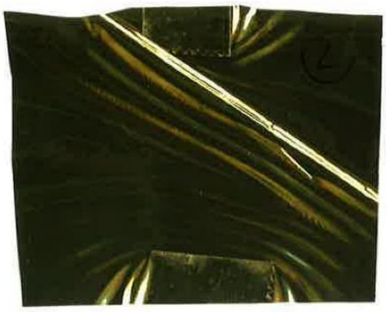

Transmission:

MRT Rescue Blanket – double layer silver

Minimal Transmission (1%) for UVB
Low transmission for UVA (3%)
Little transmission for visible light (1%) → sufficient blackout

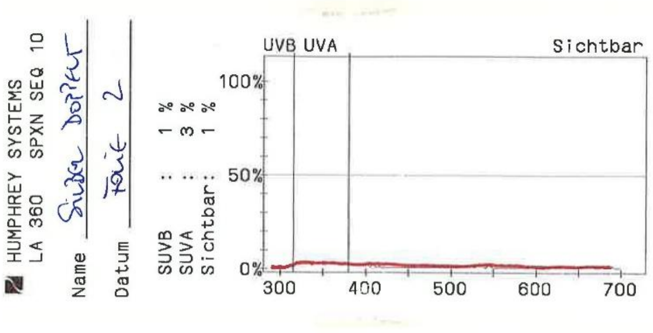

MRT Rescue Blanket – double layer silver

Results are comparable regardless whether exposed surface was silver or gold!

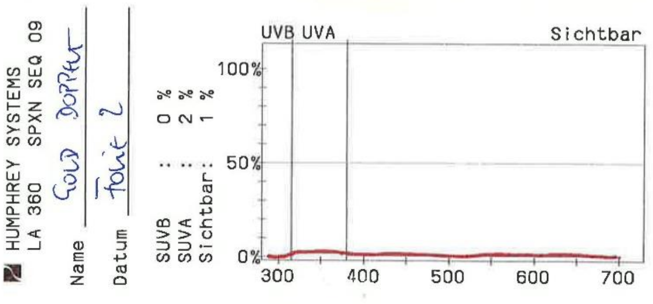

Conclusion

ARC Rescue Sheet revealed lower transmission for UV and stronger blackout in all runs compared to MRT Rescue Blanket
